# Targeting the PI3K/AKT/mTOR pathway in epithelial ovarian cancer, therapeutic treatment options for platinum-resistant ovarian cancer

**DOI:** 10.20517/cdr.2021.05

**Published:** 2021-04-14

**Authors:** Natasha Rinne, Elizabeth L. Christie, Anastasia Ardasheva, Chun Hei Kwok, Nikita Demchenko, Caroline Low, Catherine Tralau-Stewart, Christina Fotopoulou, Paula Cunnea

**Affiliations:** ^1^Department of Surgery & Cancer, Imperial College London, Hammersmith campus, London W12 0NN, UK.; ^2^Peter MacCallum Cancer Centre, East Melbourne, Victoria 3002, Australia.; ^3^Department of Metabolism Digestion & Reproduction, Imperial College London, London W12 0NN, UK.; ^4^Takeda Academic Innovation, Center for External Innovation, Takeda California, San Diego, CA 92121, USA.

**Keywords:** Ovarian cancer, high-grade serous, PI3K/AKT/mTOR pathway, chemotherapy resistance, inhibitors, therapeutics

## Abstract

The survival rates for women with ovarian cancer have shown scant improvement in recent years, with a 5-year survival rate of less than 40% for women diagnosed with advanced ovarian cancer. High-grade serous ovarian cancer (HGSOC) is the most lethal subtype where the majority of women develop recurrent disease and chemotherapy resistance, despite over 70%-80% of patients initially responding to platinum-based chemotherapy. The phosphoinositide 3-kinase (PI3K)/protein kinase B (AKT)/mammalian target of rapamycin (mTOR) signaling pathway regulates many vital processes such as cell growth, survival and metabolism. However, this pathway is frequently dysregulated in cancers including different subtypes of ovarian cancer, through amplification or somatic mutations of phosphatidylinositol-4,5-bisphosphate 3-kinase catalytic subunit alpha (*PIK3CA*), amplification of *AKT* isoforms, or deletion or inactivation of *PTEN*. Further evidence indicates a role for the PI3K/AKT/mTOR pathway in the development of chemotherapy resistance in ovarian cancer. Thus, targeting key nodes of the PI3K/AKT/mTOR pathway is a potential therapeutic prospect. In this review, we outline dysregulation of PI3K signaling in ovarian cancer, with a particular emphasis on HGSOC and platinum-resistant disease. We review pre-clinical evidence for inhibitors of the main components of the PI3K pathway and highlight past, current and upcoming trials in ovarian cancers for different inhibitors of the pathway. Whilst no inhibitors of the PI3K/AKT/mTOR pathway have thus far advanced to the clinic for the treatment of ovarian cancer, several promising compounds which have the potential to restore platinum sensitivity and improve clinical outcomes for patients are under evaluation and in various phases of clinical trials.

## INTRODUCTION

Ovarian cancer is currently the sixth most common cause of cancer death in the UK, with approximately 4100 deaths recorded in 2017^[1]^, and worldwide is the second leading cause of deaths from gynecological malignancies in westernized countries^[2]^. The overall survival rates for women with ovarian cancer have seen little improvement over the 30 years, despite advances in surgical techniques, imaging technologies, and introduction of new targeted therapies, e.g., PARP inhibitors and anti-angiogenics such as bevacizumab. Due to the lack of reliable biomarkers and the vague symptom profile of ovarian cancer, over 70% of patients will present with advanced stage disease (FIGO III or IV). Current standard-of-care for patients with ovarian cancer involves cytoreductive surgery and platinum-based chemotherapy usually in combination with a taxane. However, many patients will ultimately relapse and eventually develop chemotherapy-resistant disease, at which point treatment options are entirely palliative.

The most prevalent type of ovarian cancer, epithelial ovarian cancer (EOC), is categorized into different histological subtypes: serous (high or low grade), clear cell, endometrioid and mucinous. The classical dualistic classification of EOC into two types (I and II)^[3]^ has been revised and expanded in recent years to take into account numerous molecular and histological studies, which have provided new important insights into EOC^[4]^, and the updated WHO^[5,6]^ and FIGO classifications^[7,8]^. Type I tumors, which account for approximately 25% of all EOC, encompass the subtypes low grade serous ovarian cancer (LGSC), endometrioid, mucinous, clear cell, and the rare Brenner tumor subtype. They are largely detected at an earlier stage (FIGO I-II), have high genomic stability, usually *p53* wild-type, characterized by different mutations: LGSC (*KRAS*,* BRAF*), endometrioid (*PTEN*, *PI3KCA*), mucinous (*KRAS*,* p53*), and clear cell (*PI3KCA*, *ARID1A*), and are relatively resistant to platinum-based chemotherapy^[9]^. The more common Type II tumors are generally diagnosed at an advanced stage (FIGO III-IV), while they are initially more responsive to platinum-based chemotherapy, tend to recur and become resistant to chemotherapy, and are responsible for the majority of deaths from EOC. Subtypes of Type II include high-grade serous ovarian cancer (HGSOC), high-grade endometrioid, undifferentiated cancers and carcinosarcomas. Type II cancers are typified by high genomic instability, a near 100% *p53* mutation rate, defects in homologous recombination repair, mutations in *BRCA1* or *2*, and extensive copy number aberrations^[4]^.

HGSOC accounts for around 75% of EOC cases. The average 5-year survival rate for HGSOC is less than 40%, approximately 20% of patients do not respond to initial primary treatment efforts^[10]^ and many of the remaining patients will relapse, acquiring resistance to platinum chemotherapy^[11]^. The complete set of mechanisms giving rise to platinum resistance in HGSOC and how they cooperate have not yet been fully elucidated. Genomic analyses of HGSOC revealed widespread clonal diversity exists before chemotherapy treatment, and more recent analyses of tumors collected at relapse have identified different mechanisms of acquired resistance to platinum chemotherapy^[12-16]^. Different models of evolution of resistance to platinum chemotherapy have been proposed (reviewed in Rottenberg *et al*.^[17]^). Broadly, one model proposes the presence of genomically heterogeneous sub-clones in the chemo-naive state, and subsequent chemotherapy treatments preferentially select these resistant clones leading to eventual recurrence of the disease^[14,16,18]^. Another model proposes that treatments with DNA-damaging platinum chemotherapy cause mutations that give rise to resistance. Proposed mechanisms contributing to acquired platinum resistance in HGSOC involve activation of AKT signaling^[19]^, reversion of *BRCA1/2* germline mutations, loss of *BRCA1* methylation, extensive desmoplastic stroma, and overexpression of the *ABCB1* multidrug transporter^[15,20]^. A further model hypothesizes the role of cancer stem cells (CSC) and epithelial-to-mesenchymal transition (EMT) in causing relapse following platinum chemotherapy^[21]^. CSCs and mesenchymal-like cells characteristically have a low cycling rate suggesting that they could be more resistant to the standard cytotoxic treatments such as platinum that target actively proliferating cells^[22,23]^. Studies have shown a direct correlation between CSC abundance and the onset of relapse, suggesting CSCs promote chemotherapy resistance^[24-26]^. For example, expression of different biomarkers for CSCs, CD44, ALDH, CD133 and MyD88, was observed to be associated with chemotherapy resistance and poor patient outcome in EOC^[27-29]^. Recently, the transcription factor NFATC4 (nuclear factor of activated T cells cytoplasmic 4) was identified as a regulator of quiescence in ovarian cancer, enriched in ovarian CSCs and associated with chemotherapy resistance and poor prognosis^[30]^. Using a model of ovarian malignant ascites in a heterospheroid assay of CSCs and carcinoma-associated mesenchymal stem/stromal cells, Raghavan *et al*.^[28]^ demonstrated that PDGF (platelet-derived growth factor) signaling in these heterospheroids significantly increased stemness, metastatic potential and platinum resistance of CSCs. TWIST1 was suggested to be a regulator of EOC stemness through controlling stem cell differentiation via regulation of miR-199a and miR-214^[31-33]^. Furthermore, increased TWIST1 expression in CSCs has been shown to promote their differentiation into mesenchymal cells with CSC-like properties and capacity for migration^[32,34]^. Profiling of HGSOC tumors using the Oxford Classic classifier for molecular stratification of tumors demonstrated that EMT-high tumors were associated with poor survival and linked to immunosuppression^[35]^. However, the mechanisms of CSC and EMT-associated platinum resistance require further investigation to translate targeting of these CSC populations into improved treatment and outcomes of patients with platinum-resistant ovarian cancer.

This review aims to provide an overview of dysregulation of the phosphoinositide 3-kinase (PI3K)/protein kinase B (AKT)/mammalian target of rapamycin (mTOR) pathway in epithelial ovarian cancer, with a particular focus on the HGSOC subtype and chemotherapy resistant disease, and the potential targeting of the pathway as a therapeutic option for patients with ovarian cancer. We will present pre-clinical evidence and clinical trial outcomes for inhibitors targeting different nodes of the PI3K/AKT/mTOR pathway, and highlight past and upcoming clinical trials for ovarian cancers.

### PI3K/AKT/mTOR signaling pathway overview

**Figure 1:**
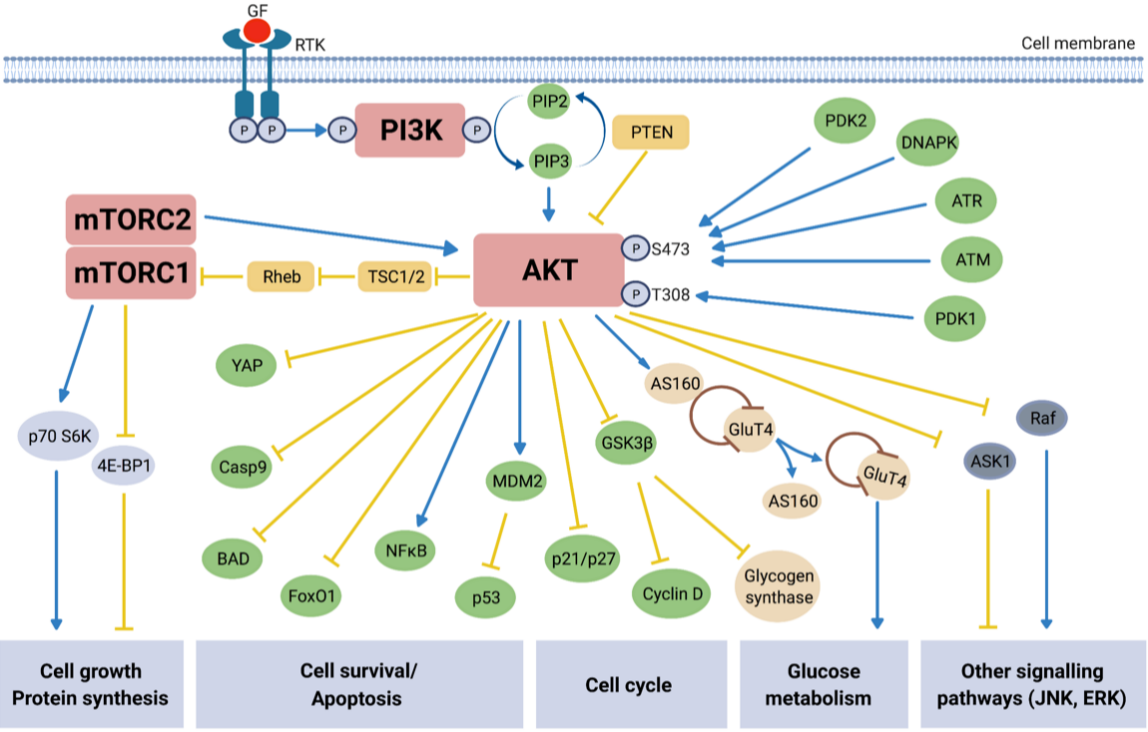
Overview of PI3K/AKT/mTOR signaling pathway. Activation (blue arrows) of growth factor (GF) receptor tyrosine kinase (RTK), resulting in autophosphorylation on tyrosine residues, recruits Phosphatidyl-inositol-3-kinase (PI3K) to the cell membrane. Direct binding of PI3K to the tyrosine residues causes activation via the PI3K catalytic subunit^[42]^. Activated PI3K in turn phosphorylates secondary messenger phosphatidylinositol-3,4,5-bisphospate (PIP2) which converts to phosphatidylinositol-3,4,5-triphosphate (PIP3). PIP3 is responsible for the recruitment of the protein kinase AKT to the cell surface where its subsequent activation/inhibition by multiple molecules leads to involvement in numerous downstream signaling pathways^[43,44]^. AKT is activated via phosphorylation at two key residues: S473 and T308^[40]^. Protein serine/threonine kinase-3’-PDK1 and PDK2, recruited to and activated at the cell surface are responsible for activating AKT, along with protein kinases ATM and ATR, and HSP90. DNA-PK, a nuclear serine/threonine kinase essential for non-homologous end-joining (NHEJ) repair, activates AKT via phosphorylation at S473 in response to cisplatin-induced DNA damage in platinum resistant EOC cells^[19]^. PTEN, tuberous sclerosis protein 1 (TSC1) and TSC2 are the main negative regulators (yellow arrows) of the pathway, with phosphorylation of TSC2 by AKT releasing the inhibitory effect on mTORC1 via the GTP-binding protein Rheb^[42]^. mTORC1 activates p70S6K and inhibits 4E-BP1, resulting in protein synthesis and cell growth, and mTORC2 activates AKT itself; overall mTOR activation leads to cell growth and survival. Inactivation of pro-apoptotic molecules YAP, Procaspase 9 (Casp9) and BAD, as well as inhibiting Forkhead transcription factors [e.g., Forkhead box protein O1 (FoxO1)] result in increased cell survival^[45,46]^. AKT is an essential part of the insulin signaling pathway. Activated in response to insulin stimulation, AKT causes Glucose Transporter 4 (GLUT4) to translocate to the cell surface, facilitating glucose uptake. Additionally, the inhibition of glycogen synthase kinase 3 beta (GSK-3β) by AKT increases glycogen production^[47]^. The role of AKT in glucose metabolism is again apparent through its inhibition of FoxO1, which suppresses hepatic glucose production^[48]^. The PI3K/AKT/mTOR signaling pathway includes points of cross-regulation and communication with other common signaling pathways, e.g., JNK and RAS-ERK. The AKT substrate c-RAF is activated by RAS and initiates a kinase cascade leading to ERK activation^[49]^. The phosphorylation of c-RAF by AKT inhibits its activity and subsequent downstream activity in the RAS/RAF/MEK/ERK signaling cascade known for its role in apoptosis and cell differentiation^[50]^. AKT involvement with the JNK pathway, a stress- and nutrient-response pathway, directly modifies activation of target genes, many of which are involved in adaptations to extra-cellular stresses^[51]^.

Extensive characterization of the PI3K/AKT/mTOR signaling pathway over the last two decades has led to a much greater understanding of the molecular mechanisms underlying this pathway’s regulation of essential cellular processes such as cell growth, survival, proliferation, angiogenesis, metabolism, transcription, and translation^[36]^. The classical mechanisms behind the canonical PI3K/AKT/mTOR pathway activation and its functions are described in Figure 1. Phosphoinositide 3-kinases (PI3Ks) are lipid kinases which are major downstream effectors of G protein coupled receptors (GPCRs) and receptor tyrosine kinases (RTKs). There are three classes of PI3K (classes I, II, III), in which class I is comprised of class IA PI3K (α, β, δ) and class IB PI3K (γ). Each sub-class of PI3K class IA or IB is activated by receptors RTKs or GPCRs, respectively. Class IA PI3Ks are composed of a p85 regulatory subunit of which there are five variants and a p110 catalytic subunit^[37,38]^. There are three class IA p110 isoforms (α, β, δ) expressed by the genes phosphatidylinositol-4,5-bisphosphate 3-kinase catalytic subunit alpha (*PIK3CA*),* PIK3CB* and *PIK3CD*, respectively, of which *PIK3CA *is the most frequently mutated in multiple cancer types, including EOC^[37,39]^.

In brief, the pathway becomes activated following phosphorylation of RTKs in response to extracellular signals, thus activating PI3K (p85, p110)^[36]^. PI3K converts phosphoinositide 4,5-biphosphate (PIP2) to phosphatidylinositol (3,4,5)-triphosphate (PIP3). PIP3 activates AKT (protein kinase B) via phosphoinositide-dependent kinase-1 (PDK1)-mediated phosphorylation at the Threonine 308 (T308) site of AKT and is recruited to the cell surface. PIP3 is later dephosphorylated by the tumor suppressor and negative regulator of the pathway phosphatases and tensin homolog (PTEN) back to PIP2. The full activation of AKT is achieved with participation of the mammalian target of rapamycin complex 2 (mTORC2) complex, which phosphorylates AKT at the Serine 473 (S473) site^[40]^. Protein kinases ATM (ataxia-telangiectasia mutant), ATR (ataxia-telangiectasia and Rad3 related) and DNA-PK (DNA-dependent protein kinase), and HSP90 (heat shock protein 90) can also activate AKT via phosphorylation at S473, demonstrating the diversity of cellular contexts in which AKT plays a role^[19,41]^.

### PI3K/AKT/mTOR pathway alterations in ovarian cancer

Dysregulation of the PI3K pathway is frequently observed in human cancers, comprising of amplification and somatic mutations of *PIK3CA*, deletion or inactivation of *PTEN *and amplification or mutation of *AKT* isoforms (reviewed in Janku *et al*.^[52]^). In EOC, early genomic investigations demonstrated frequencies of genetic alterations for *PIK3CA* of 4%-12% (somatic mutations) in epithelial ovarian tumors^[39,53,54]^, 20%-46% of clear cell ovarian cancers^[53,55]^, 20% of endometrioid ovarian cancers, and 2.3%-3% of HGSOC^[53,56]^. Frequent amplification of *AKT2* was identified in different ovarian cancer cohorts, for example in 18.2% of patients with HGSOC^[57]^ and 10% of patients with advanced ovarian cancer (cohort comprised of 89% HGSOC)^[58]^. Loss of *PTEN* was observed in different histological subtypes of ovarian cancer, via different mechanisms (deletion, loss-of-heterozygosity, epigenetic silencing or loss-of-function mutations)^[59-62]^. Significant advances in sequencing technologies in the last decade have further increased our understanding of the genomic dysregulation of the PI3K pathway. In 2011, the Cancer Genome Atlas (TCGA) study demonstrated that 45% of serous ovarian tumors had dysregulation of the PI3K pathway^[13]^. In particular, 7% had deletions of *PTEN*, < 1% had *PTEN* mutations, 18% had amplification or mutations of *PIK3CA*, and amplification of *AKT1* and *AKT2* was found in 3% and 6% of cases, respectively^[13]^. Dysregulated components of the PI3K pathway were also found to be linked to chemotherapy resistance in HGSOC. In the ICGC cohort of chemotherapy sensitive, refractory and resistant HGSOC tumors, inactivation of genes by disruption of transcriptional units (gene breakage) was observed for tumor suppressor genes including *PTEN,* and gene breakage was associated with acquired chemotherapy resistance^[15]^. Recently, seven copy number signatures were derived for HGSOC which may predict both overall survival and the probability of platinum-resistant relapse. Across these 7 signatures, signature exposures 4 and 6 were associated with aberrant PI3K signaling (mutation of *PIK3CA*, amplification of *AKT2* and *RICTOR*)^[63]^. Table 1 outlines the frequencies of the main genomic alterations (amplification, deletion, and mutations) observed in HGSOC in the TCGA, ICGC and BriTROC-1 cohorts^[13,15,63]^. In HGSOC, genomic amplification of *PIK3CA*, AKT isoforms (*AKT2*,* AKT3*) or *RICTOR* or deletion of *PTEN* are relatively common, whereas mutations in these genes are rare.

**Table 1:** PI3K/AKT/mTOR pathway genomic alterations for HGSOC in TCGA*, ICGC OV-AU^§^ and BriTROC-1 cohorts

**Gene**	**Alteration**	**Frequency of alteration %**		
		TCGA*	**ICGC(OV-AU)^§^**	**BriTROC-1**
*AKT1*	Amplification	2.9-5.2	2.2	
	Deletion	0-0.2		
	Mutation	0.3	3.3	
*AKT2*	Amplification	5.8-8	9.8	11.4
	Deletion	0.3-1		
	Mutation		3.3	
*AKT3*	Amplification	4.1-9.5	4.3	
	Deletion	0.2-0.4		
	Mutation	1	7.6	
*PIK3CA*	Amplification	18-29	22.8	
	Mutation	1	2.2	4.6
*PIK3CB*	Amplification	3.9-11.4	13.0	
	Mutation	0.3	1.1	2.3
*PIK3R1*	Amplification	0-0.2		
	Deletion	2-3.5		
	Mutation	2	2.2	
*PTEN*	Amplification	0.7-1.2		
	Deletion	4.5-6.1	2.2	
	Mutation	1	6.5	
*RICTOR*	Amplification	3.5-7.1	3.3	9.1
	Mutation		1.1	

Mutation types include missense mutations, truncating mutations or fusion. *TCGA studies accessed via cBioPortal; data presented involve the range of 3 studies. ^§^ICGC OV-AU cohort accessed via https://icgc.org/4cV. BriTROC-1 data accessed via^[63]^.

Several studies investigating PI3K signaling in ovarian cancer have demonstrated enhanced signaling of different nodes of the pathway and how dysregulation of the PI3K/AKT/mTOR pathway contributes to cell proliferation, migration, and chemotherapy resistance. PI3K signaling outputs were determined by quantifying p-AKT, p-p70S6K and p-GSK3β in ascites samples from patients with advanced ovarian cancer, predominately the HGSOC subtype. Significantly higher levels of p-p70S6K levels were detected in patients who did not respond to chemotherapy^[58]^. Immunohistochemical staining of tissue microarrays (TMA) of different ovarian cancer subtypes showed significantly increased staining of p-4EBP1, p-p70S6K and p-S6, in particular p-4EBP1 expression correlated with high-grade tumors and poor prognosis^[64]^. A multi-center study from the Ovarian Tumor Tissue Analysis Consortium of over 5400 patient tumors investigated PTEN loss as a putative driver in different histological subtypes of ovarian cancer^[65]^. Downregulation of cytoplasmic PTEN expression was most frequent in endometrioid and clear cell ovarian cancers and associated with longer overall survival in HGSOC. PTEN loss was demonstrated to be a frequent driver in ovarian cancer, with strong associations with expression of the Androgen, Estrogen and Progesterone hormone receptors, and CD8+ TILs (tumor infiltrating lymphocytes) in HGSOC and clear cell ovarian cancers^[65]^. In a study by Huang *et al*.^[66]^, p-AKT-S473 and p110α overexpression was demonstrated to be significantly associated with decreased survival in a cohort of 522 serous ovarian tumors. They also examined how loss of a number of PI3K-associated proteins affected vital cellular processes. Following small interfering RNA (siRNA) knockdown of *PIK3CA* in *PIK3CA*-mutant A2008 and *PIK3CA *copy-gain UPN251 cells, cell proliferation decreased by almost 50%^[66]^. The same study showed a more prominent increase of apoptosis (5.5-fold) and decrease of proliferation (51%) following knockdown of *AKT2* in the OVCAR8 cell line, known to have a copy-gain of *AKT2*, compared to the other ovarian cancer lines tested. None of the *PIK3CA, PIK3CB* or *AKT2* siRNAs induced apoptosis in normal ovarian surface epithelial cell lines^[66]^. Montero *et al*.^[67]^ aimed to establish the functional relevance of mammalian target of rapamycin complex 1 (mTORC1) and mTORC2 in epithelial ovarian cancer cell lines (OVCAR8, SKOV3, A2780, and IGROV-1) as potential clinical targets. Using siRNAs against raptor or rictor to target mTORC1 and mTORC2, respectively, demonstrated that knockdown of either protein led to a significant decrease in cell proliferation. However, knockdown of raptor/mTORC1 displayed greater inhibitory effects than of rictor/mTORC2, suggesting a more important role for mTORC1 in ovarian cancer cell proliferation^[67]^. Raptor knockdown led to decreased levels of phosphorylated ribosomal S6 kinase-S240/244 and p4E-BP1, and also an increase of p-AKT-S473, indicating a negative feedback orchestrated by mTORC1 over mTORC2. Overall, data suggested targeting of both mTORC complexes as a clinical strategy. Furthermore, as the mTOR inhibitor BEZ-235 displayed a synergistic effect with cisplatin chemotherapy *in vitro*, combining mTOR inhibitors with standard-of-care chemotherapy was proposed as a viable option for ovarian cancer patients^[67]^.

The role of AKT in chemotherapy resistance and targeting AKT as a strategy to re-sensitize chemotherapy resistant HGSOC cells to platinum-based chemotherapy has been explored. A study by Yang *et al.*^[68]^ investigated the role of AKT in caspase-independent apoptosis in cisplatin-resistant OC cells. Cisplatin treatment induced the release of apoptosis-initiating factor (AIF), a mediator of caspase-independent apoptosis, in cisplatin-sensitive cells only, and overexpression of AIF in cisplatin-resistant cells re-sensitized cells to cisplatin. Furthermore, AKT negatively regulated AIF, and downregulation of AKT re-sensitized resistant cells to cisplatin-induced AIF-dependent apoptosis, suggesting that AKT plays a significant role in chemotherapy resistance and its inhibition can re-sensitize platinum-resistant cells to cisplatin^[68]^. In concordance, research by Stronach *et al*.^[19]^ identified AKT as a potential mediator of platinum resistance in immortalized patient-acquired platinum-resistant HGSOC cell lines. Upon cisplatin treatment of platinum-resistant HGSOC cells, AKT relocated to the nucleus where it was phosphorylated at S473 by DNA-PK, inducing an AKT-dependent DNA damage response, and this activation of AKT led to an inhibition of platinum-mediated apoptosis in platinum-resistant cells. Furthermore, inhibition of AKT with the allosteric AKT inhibitor triciribine, led to a decrease in phosphorylated AKT-S473 in the presence or absence of cisplatin. Inhibition of AKT in combination with cisplatin treatment enhanced apoptotic induction in platinum-resistant HGSOC cells. Knockdown or inhibition of DNA-PK had a similar effect as triciribine treatment in platinum-resistant cells, enhancing apoptosis and decreasing accumulation of phosphorylated AKT-S473 in combination with cisplatin administration. Interestingly, targeting DNA-PK did not affect insulin-mediated activation of AKT, which is an alternative route for AKT activation upon its direct inhibition. Thus, it could be concluded that DNA-PK inhibition could target AKT without causing toxicity that is often observed when direct inhibitors of AKT are used, while still enhancing apoptosis^[19]^.

### Targeting of PI3K/AKT/mTOR pathway in ovarian cancer

Frequent activation of the PI3K/AKT/mTOR pathway in many cancers, including ovarian cancer, suggests the PI3K/AKT/mTOR pathway to be an attractive target for therapeutic intervention^[52,69]^. Following the approval of the mTORC1 inhibitors everolimus and temsirolimus over a decade ago for breast cancer and renal cell carcinoma^[70,71]^, several inhibitors of the pathway have been developed as a potential monotherapy or in combination with other therapeutics (e.g., olaparib^[72]^, and bevacizumab^[73]^) or chemotherapy drugs (e.g., carboplatin^[74]^, and docetaxel^[75]^). Inhibitors targeting different nodes of the PI3K/AKT/mTOR pathway can be broadly categorized into four groups: PI3K inhibitors, mTOR inhibitors, dual PI3K/mTOR inhibitors, and AKT inhibitors. Clinical trials have been established for several compounds from each class of pathway inhibitor involving patients with ovarian cancer including platinum-refractory or resistant ovarian cancer cohorts. Tables 2 and 3 outline examples of PI3K/AKT/mTOR pathway inhibitors from each group in clinical trials that have been completed or recently completed and awaiting results [Table 2], or active and/or recruiting [Table 3] for ovarian cancer or solid malignancies including ovarian cancer patients. While a number of PI3K pathway inhibitors have been approved for use in patients by the Food and Drug Administration (FDA) for other cancer types, no compounds have yet progressed to clinical use for ovarian cancer. The majority of trials for PI3K/AKT/mTOR pathway inhibitors specifically targeting ovarian cancer cohorts are early-phase trials including several first-in-human, establishing pharmacokinetics (PK) and pharmacodynamic (PD) profiles, dose escalation, and combination studies. Enrolment based on a biomarker profile of different dysregulated nodes (e.g., *PIK3CA* mutation or amplification, loss of* PTEN*) of the pathway is a prerequisite in only a small number of the trials highlighted in Tables 2 and 3. However, most studies report including retrospective profiling of trial samples for potential biomarkers of clinical response. In the following sections, we will highlight the findings of some of the key pre-clinical studies and clinical trials for inhibitors of the PI3K/AKT/mTOR pathway, according to inhibitor groups.

**Table 2:** Completed or ongoing clinical trials for inhibitors of the PI3K/AKT/mTOR pathway according to highest phase reached for trials including ovarian cancer patients, indication, monotherapy or combination therapy, clinical trials and publication references

**Compound/generic name**	**Mechanism of action**	**Status/highest phase**	**Indication (± biomarker enrolment criteria)**	**Monotherapy/combination therapy**	**Clinical trials reference**	**Clinical trial outcome/reference**
**PI3K**						
Buparlisib BKM120	Class I pan-PI3K inhibitor	Phase II	Advanced solid malignancies incl. ovarian cancer patients with PI3K-activated tumors	Monotherapy	NCT01833169	ORR 1.4% (*n *= 2); CBR 15.1% (*n* = 22)^[94]^
CH5132799	Class I PI3K, in particular PI3Kα	Phase I	Advanced solid malignancies incl. ovarian cancer patients; dose escalation study	Monotherapy	NCT01222546	MTD of 48mg BID; *n *= 1 PR by GCIG CA125^[91]^
Pictilisib GDC-0941	Class I pan-PI3K inhibitor	Phase I	Advanced solid malignancies incl. ovarian cancer patients; first-in-human dose escalation study	Monotherapy	NCT00876122	Well tolerated; 3% *n *= 2 PR by RECIST or GCIG CA125. RP2D of 330mg QD^[95]^
PX-866	Class I pan-PI3K inhibitor	Phase I	Advanced solid malignancies incl. ovarian cancer patients; dose escalation study	Docetaxel	NCT01204099	PR 6% *n *= 2; SD 63% *n *= 22; PD 31% *n *= 11. Median PFS 73.5 days (range: 1-569). RP2D of 8mg^[75]^
Alpelisib BYL-719	PI3Kα	Phase Ib	Recurrent ovarian, fallopian tube, or primary peritoneal cancer of HGS histology or recurrent TNBC; ± known germline *BRCA* mutations; dose escalation and expansion trial	Olaparib	NCT01623349	MTD and RP2D of 200mg alpelisib QD + olaparib 200mg BID; EOC pts PR 36% (*n *= 10), SD 50% (*n *= 14) by RECIST^[93]^
IPI-549	PI3Kγ	Phase I/Ib	Advanced solid tumors; first in human study	Nivolumab	NCT02637531	MTD not reached. PR *n *= 2 at 8wk assessment. RP2D IPI-549 40 mg QD + nivo 240 mg Q2W^[96]^
TAK117 Serabelisib	PI3Kα	Phase Ib (ongoing)	Advanced ovarian, endometrial, or breast cancer	Sapanisertib Paclitaxel	NCT03154294	ORR 46% in 13 evaluable pts. CBR of 69%; PFS = 10 months. CR *n *= 2 pts^[97]^
**PI3K/mTOR**						
Apitolisib GDC-0980 G-038390	Class I pan-PI3K/mTOR inhibitor	Phase I	Advanced solid tumors incl. ovarian; first in human	Monotherapy	NCT00854152	PR *n *= 10 pts by RECIST; RP2D of 40mg QD 28/28 schedule^[98]^
Bimiralisib PQR309	Dual PI3K mTORC1/2 inhibitor	Phase I	Advanced solid tumors (SAKK 67/13), incl. ovarian patients; first-in human, dose escalation trial	Monotherapy	NCT01940133	MTD and RP2D is 80 mg QD; PR *n *= 1, SD > 16 weeks *n *= 1 by RECIST^[99]^
SF1126	Pan-PI3K/mTORC prodrug	Phase I	Advanced or metastatic tumors incl. ovarian cancer patients; first-in human	Monotherapy	NCT00907205	SD in 58% (*n *= 19) of evaluable pts. MTD not reached^[100]^
BGT226	Class I pan-PI3K/mTOR inhibitor	Phase I/II	Advanced solid malignancies incl. ovarian cancer patients; dose escalation study	Monotherapy	NCT00600275	MTD 125mg/day TIW; SD 30% (*n *= 17); SD ≥ 16 weeks 16% (*n *= 9) by RECIST^[101]^
Gedatolisib PKI-587 PF-05212384	Class I pan-PI3K/mTOR inhibitor	Phase I	Solid tumors, incl. ovarian cancer patients; first-in human	Monotherapy	NCT00940498	MTD of 154mg QW; PR 2.5% (*n *= 2); SD > 6mths of 10% (*n *= 8) by RECIST^[102]^
LY3023414 Samotolisib	ATP competitive class I PI3K inhibitor, mTOR and DNA-PK	Phase Ib	Advanced or metastatic tumors incl. ovarian cancer patients	Prexasertib	NCT02124148	No results posted to date for LY3023414 and Prexasertib combination
XL765 SAR245409	Dual inhibitor of mTOR/PI3K	Phase II	Patients with previously treated unresectable low grade ovarian cancer	Pimasertib	NCT01936363	ORR of 9.4% in combination arm; median PFS of 7.23 mths pimasertib alone and 9.99 mths pimasertib + SAR245409^[103]^
**mTOR**						
Temsirolimus CCI-779	mTORC1 inhibitor	Phase II	Ovarian Clear Cell Cystadenocarcinoma, Stage III Ovarian Cancer, Stage IV Ovarian Cancer	Carboplatin Docetaxel Paclitaxel	NCT01196429	US/Korea cohort CR 31%, PR 23%; Japan cohort CR 6%, PR 65%^[104]^
Everolimus RAD-001	mTORC1 inhibitor	Phase II	Recurrent ovarian, peritoneal, and Fallopian tube cancer (RADBEV trial)	Bevacizumab	NCT01031381	24% of pts progression-free at 6mths. CR *n *= 1; PR *n *= 6 pts; SD *n *= 35 pts^[73]^
Sirolimus	mTORC1 inhibitor	Phase II	Stage II-IV ovarian epithelial, Fallopian tube, or primary peritoneal cavity cancer	Vaccine Therapy	NCT01536054	No results posted to date
BI-860585	mTORC1/mTORC2 inhibitor	Phase I	Advanced and/or metastatic solid tumors incl. ovarian cancer patients	Exemestane Paclitaxel	NCT01938846	BI 860585 + exemestane PR *n *= 4; BI 860585 + paclitaxel PR *n *= 4, CR *n *= 1^[105]^
CC-115	mTOR/DNA-PK	Phase I	Advanced solid tumors and hematologic malignancies; first in human dose escalation and expansion study	Monotherapy	NCT01353625	MTD reached in 5 tumour types; SD in ovarian pts (*n *= 2)^[106]^
**AKT**						
Uprosertib GSK-2141795	Pan-AKT isoform inhibitor	Phase I	Recurrent or persistent ovarian cancer	Monotherapy	NCT01266954	CBR of 27% by RECIST; 30% by CA125 GCIG^[107-109]^
Afuresertib GSK-2110183	Pan-AKT isoform inhibitor	Phase Ib/II	Recurrent platinum-resistant ovarian cancer	Carboplatin Paclitaxel	NCT01653912	ORR of 32% by RECIST, 52% by GCIG CA125 Median PFS was 7.1 mths^[74]^
MK-2206	Pan-AKT isoform inhibitor	Phase II	Recurrent platinum-resistant ovarian, fallopian tube, or peritoneal cancer	Monotherapy	NCT01283035	SD in 4 of 5 evaluable pts; *n *= 1 SD for 19wks; *n *= 1 SD for 10.8 wks. Median PFS estimated 19wks^[110]^
Capivasertib AZD5363	Pan-AKT isoform inhibitor	Phase I	Recurrent endometrial, TNBC, and ovarian, primary peritoneal, or fallopian tube cancer. Biomarker enrolment: germline *BRCA1/2*-mutant, or *BRCA1/2* wild-type cancers harboring somatic DDR or PI3K–AKT pathway alterations	Olaparib	NCT02338622	44.6% (25/56) evaluable pts had CBR (RECIST CR/PR or SD ≥ 4 months)^[72]^
ARQ-092 Miransertib mesylate	Pan-AKT isoform inhibitor	Phase I (terminated)	Advanced, inoperable metastatic and/or recurrent solid tumors, ovarian or endometrial cancer. Enrolment criteria for endometrial cancer: *AKT1, PIK3CA, or PIK3R1* mutations with or without *PTEN* deficiency	Carboplatin Paclitaxel Anastrozole (endometrial cancer)	NCT02476955	Responses in EC 4/8 pts: CR *n *= 1 (confirmed), PR *n *= 3 (unconfirmed)^[111]^
PTX-200 Triciribine	AKT inhibitor	Phase I	Recurrent or persistent, platinum-resistant epithelial ovarian, fallopian tube or primary peritoneal carcinoma	Carboplatin	NCT01690468	Study terminated due to change in strategic focus
Perifosine D-21266	Pan-AKT isoform inhibitor	Phase I	Recurrent ovarian cancer; in combination with Doxetaxel	Doxetaxel	NCT00431054	Median PFS of 1.9 mths; OS of 4.5 mths^[112]^

Data collated for table from database searches of NCI clinical trials database (clinicaltrials.gov), literature searches (pubmed.com) and Cortellis Drug Discovery website (cortellis.com). TNBC: Triple negative breast cancer; EC: endometrial cancer; DDR: DNA damage repair; MTD: maximum tolerated dose; ORR: objective response rate; CBR: clinical benefit rate; CR: complete response; PR: partial response; PD: progressive disease; SD: stable disease; RP2D: recommended phase 2 dose; QD: every day; BID: twice daily; TIW: 3 times per week; Q2W: once every 2 weeks; GCIG: gynecological cancer intergroup; RECIST: response evaluation criteria in solid tumors.

**Table 3:** Active or currently recruiting clinical trials for inhibitors of the PI3K/AKT/mTOR pathway according to the highest phase including ovarian cancer patients, indication (± biomarkers), monotherapy or combination therapy, clinical trials and year opened

**Compound/generic name**	**Mechanism of action**	**Status/highest phase**	**Indication (± biomarker enrolment criteria)**	**Monotherapy/combination therapy**	**Clinical trials reference**	**Year opened**
**PI3K**						
BGB-10188	PI3Kδ inhibitor	Phase I/II (recruiting)	Mature B-Cell malignancies and combination with Tislelizumab in patients with solid tumors (including ovarian cancer)	Tislelizumab	NCT04282018	2020
CYH-33	PI3Kα inhibitor	Phase I (recruiting)	Patients with DDR gene mutations and/or *PIK3CA* mutations, in patients who progressed on prior PARP inhibitor, and patients with recurrent HGSOC, fallopian tube, or primary peritoneal cancer who are platinum resistant or refractory	Olaparib	NCT04586335	2020
Linperlisib YY-20394	PI3Kδ inhibitor	Phase I (recruitment to start)	Advanced solid tumors	Monotherapy	NCT04049929	2019
Eganelisib IPI-549	PI3Kγ inhibitor	Phase I/Ib (recruiting)	Advanced metastatic TNBC or ovarian cancer	Etrumadenant; PLD; Nab-paclitaxel	NCT03719326	2018
Copanlisib BAY-806946	Class I pan-PI3K inhibitor	Phase II (recruiting)	Recurrent endometrial and recurrent ovarian, primary peritoneal, or Fallopian tube cancer or *BRCA* mutant ovarian cancer	Niraparib	NCT03586661	2018
TAK117 Serabelisib	PI3Kα	Phase Ib (recruiting)	Patients with advanced ovarian, endometrial, or breast cancer	Sapanisertib Paclitaxel	NCT03154294	2017
Pictilisib or Taselisib	Class I pan-PI3K inhibitor	Phase Ib (active)	Advanced solid tumors and breast cancer. *PI3KCA* mutant tumors. PIPA trial	Palbociclib	NCT02389842	2015
**mTOR**						
Everolimus	mTORC1 inhibitor	Phase I (recruiting)	Advanced ovarian (platinum resistant or refractory) and breast cancer (triple negative or hormone receptor positive only)	Niraparib	NCT03154281	2017
Sirolimus	mTORC1 inhibitor	Phase IV (recruiting)	Refractory Solid Tumors (*PIK3CA* mutation, *PIK3CA* amplification, PIK3CA-AKT pathway aberration)	Monotherapy	NCT02688881	2017
**AKT**						
Afuresertib	Pan-AKT isoform inhibitor	Phase II (recruiting)	Platinum-resistant ovarian cancer (PROFECTA-II)	Paclitaxel	NCT04374630	2020
Ipatasertib GDC-0068	Pan-AKT isoform inhibitor	Phase I/II (active)	Advanced breast cancer, ovarian cancer (HGSOC or endometrioid or clear cell epithelial ovarian, Fallopian tube, or primary peritoneal cancer), or prostate cancer	Rucaparib	NCT03840200	2019
ARQ751	Pan-AKT isoform inhibitor	Phase Ib (active)	Solid tumors with *PIK3CA/AKT/PTEN* mutations	Paclitaxel Fulvestrant	NCT02761694	2016

Data collated for table from database searches of NCI clinical trials database (clinicaltrials.gov), literature searches (pubmed.com) and Cortellis Drug Discovery website (cortellis.com). TNBC: Triple negative breast cancer; DDR: DNA damage repair; PLD: pegylated liposomal doxorubicin; Nab paclitaxel: nanoparticle albumin-bound paclitaxel.

#### PI3K inhibitors

Commonly PI3K isoforms are categorized into three classes: class I, class II and class III PI3Ks^[76]^. Class I PI3Ks are the main isoforms targeted in cancer drug development^[37]^. PI3K inhibitors can be subdivided in to those that target one or two specific isoforms (α, β, γ, δ), and pan-PI3K inhibitors, which target the catalytic activity of all four PI3K class I isoforms^[69,77]^. While theoretically, targeting all four isoforms may appear to be a useful therapeutic strategy, this may lead to an increased risk of off-target effects and toxicity for patients. Consequently, the clinical development of the majority of pan-PI3K inhibitors has been severely limited due to poor outcomes and safety concerns such as increased drug-related toxicities, including hyperglycemia, gastrointestinal and neuropsychiatric side-effects^[78]^. Class I isoforms have different activation mechanisms which suggest that each isoform has distinct biological functions^[76,79]^, therefore targeting individual isoforms may have a wider therapeutic benefit.

The first generation of pan-PI3K inhibitors developed, LY294002 and wortmannin, were shown to exert anti-cancer effects in a number of pre-clinical ovarian cancer cell models^[36,80,81]^. Hu *et al*.^[82]^ demonstrated that LY294002 could inhibit tumor formation *in vivo* in a mouse model of ovarian cancer, and could inhibit proliferation of OVCAR-3 platinum-resistant ovarian cancer cells *in vitro*. Similarly Fekete *et al*.^[83]^ showed that LY294002 is able to enhance the cytotoxic effects of the chemotherapeutic agents carboplatin and paclitaxel in the platinum-resistant ovarian cancer cell lines SKOV-3 and IGROV-1. In addition, the pan-PI3K inhibitor wortmannin has been shown both to enhance apoptosis in platinum-resistant ovarian cancer cells (A2780cis)^[84]^, and to sensitize ovarian cancer cells to cisplatin in Caov3 mouse models^[85]^.

Buparlisib (BKM120) is one of the most widely studied pan-PI3K inhibitors. Bendell *et al*.^[86]^ conducted a Phase I study looking at BKM120 as single agent treatment in patients with advanced solid tumors including ovarian cancer; one patient out of 35 achieved partial response and 16 had a stable response for more than six weeks. Buparlisib has also been tested in combination with other agents in a number of ovarian cancer trials, for example a Phase II trial was performed administering buparlisib in combination with the MEK1/2 inhibitor trametinib where ovarian cancer patients were shown to have a 29% overall response rate and median progression-free survival of seven months^[87]^. *In vitro* buparlisib has been tested in combination with the PARP inhibitor olaparib and shown to effectively inhibit proliferation, survival and invasion in the *PIK3CA* mutant ovarian cancer cell lines SKOV-3, IGROV-1, and HEYA-8^[88]^. A dose escalation Phase I trial was recently conducted testing buparlisib in combination with olaparib in a cohort of 69 patients including 46 ovarian cancer patients, showing a 29% response rate irrespective of platinum sensitivity^[89]^.

Other pan-PI3K inhibitors (XL147, GDC0941, CH5132799, and PX-866) have been under clinical investigation in Phase I-II trials [Table 2]^[90]^. For example, the pan-PI3K inhibitor PX-866 has been tested in combination with docetaxel in a Phase I multi-center study in 43 patients with advanced solid tumors, including 5 patients with ovarian cancer. In the trial overall, 6% of patients achieved a partial response, 63% of patients had stable disease, and 31% of patients had disease progression^[75]^. A Phase I trial in patients with metastatic cancer by Blagden *et al*.^[91]^ studying toxicity, PK and PD of CH5132799, a pan-PI3K inhibitor particularly inhibiting PI3Kα, revealed one patient with *PIK3CA*-mutant clear cell carcinoma of the ovary achieving a partial response (GCIC CA125 criteria) (NCT03767335).

Isoform-specific inhibitors have been developed to reduce off-target effects and the cumulative toxicity observed with pan-PI3K isoform inhibition^[52,69]^. Pre-clinical investigations of a considerable number of isoform-specific PI3K inhibitors in solid tumors and leukemia, e.g., alpelisib (PI3Kα), SAR260301 (PI3Kβ), IPI-549 (PI3Kγ), and idelalisib (PI3Kδ)^[36,52,92]^, have led to Phase I trials, but limited progress has been achieved beyond Phase I with these inhibitors^[52,69]^. The efficacy of alpelisib was recently evaluated in a Phase I trial in combination with olaparib in patients with recurrent ovarian cancer or TNBC, based on pre-clinical data showing that treatment with PI3K inhibitors may sensitize homologous recombination repair-proficient EOC tumors to PARP inhibition. The EOC patients (*n *= 28) within the trial cohort achieved 36% partial response and 50% of patients had stable disease, with no unexpected toxic effects observed (grade 3-4 adverse events observed in low numbers of patients), showing that this synergistic combination deserves further clinical investigation^[93]^. There are a number of actively recruiting Phase I clinical trials looking at both pan- and isoform-specific PI3K inhibitors in advanced solid tumors and in particular recurrent ovarian cancer [Table 3], as monotherapy or in combination with PARP inhibitors (olaparib or niraparib), or immunotherapy agents (tislelizumab). While toxic side effects and low clinical efficacy have limited the advancement of many pan-PI3K inhibitors, PI3K isoform specific inhibitors may provide a better alternative with less off-target effects and adverse events for patients.

#### mTOR inhibitors

The multi-protein complexes, mTOR complex 1 (mTORC1) and mTOR complex 2 (mTORC2) comprise the serine threonine protein kinase mTOR. Inhibitors of mTOR are either allosteric targeting either mTORC1 or mTORC2, or target both mTORC1/mTORC2 (non-allosteric/catalytic inhibitors)^[52]^. A number of *in vitro* studies have investigated allosteric and catalytic mTORC inhibitors as single agents or in combination with other agents in platinum-resistant ovarian cancer among other solid tumors, with promising findings. Pre-clinical *in vitro* studies with the allosteric mTORC1 inhibitor rapamycin (sirolimus), demonstrated that treatment of the platinum-resistant SKOV3 ovarian cancer cell line with sirolimus enhanced cisplatin-mediated apoptosis^[113]^. Likewise, Mabuchi *et al*.^[114]^ showed that treatment with another allosteric inhibitor, everolimus (RAD001), enhanced cisplatin-induced apoptosis in platinum-resistant ovarian cancer cell lines (SKOV3 and OVCAR-10) with high AKT/mTOR activity, and inhibition of tumor growth and angiogenesis in mouse SKOV3 xenograft models.

The first PI3K/AKT/mTOR pathway inhibitors developed targeting mTORC1, everolimus and temsirolimus, are the most widely investigated mTOR inhibitors for ovarian cancer. Several studies have progressed to Phase I/II clinical trials, showing that mTOR inhibitors exhibit more promising results in combination with anti-angiogenics and/or chemotherapeutic agents than as a monotherapy^[36]^. A Phase II study conducted in 54 patients with persistent/recurrent epithelial ovarian cancer/primary peritoneal cancer, tested temsirolimus as a weekly intravenous (IV) single agent, showing modest activity and found 24% patients had progression-free survival (PFS) > 6 months (median 3.1 months)^[115]^. A Phase I study by Kollmannsberger *et al*.^[116]^ administered temsirolimus in combination with carboplatin and paclitaxel in six ovarian cancer patients, resulting in disease stabilization (*n *= 3) or partial response (*n *= 3) in patients^[117]^. A further Phase Ib trial administered tesmirolimus in combination with pegylated liposomal doxorubicin in a cohort of patients with advanced breast, endometrial and ovarian cancers. Patients with ovarian cancer demonstrated durable partial responses of over 10 months (*n *= 2) or stable disease (median 6.4 months) (*n *= 2)^[118]^.

Clinical trials have been established to determine the efficacy of mTOR inhibitors in combination with anti-angiogenic agents. Following on from a dose-finding Phase I clinical trial in solid malignancies^[119]^ and two Phase II trials in refractory metastatic colorectal cancer^[120]^ and renal cell carcinomas^[121]^, a recent study by Taylor *et al*.^[73]^ investigated the efficacy of everolimus and bevacizumab in a Phase II trial in recurrent ovarian, peritoneal and fallopian tube cancers. The oral administration of everolimus in combination with the IV administration of bevacizumab every two weeks resulted in 24% of patients (95%CI: 16.67%-42.71%) being progression-free at six months^[73]^.

Work to date on mTOR inhibitors has mainly focused on the allosteric mTORC1 or 2 inhibitors, but dual non-allosteric inhibition of mTORC1/mTORC2 has been demonstrated to overcome the feedback loop activation of PI3K and AKT in the pathway observed following inhibition with allosteric mTORC1 inhibitors, and therefore non-allosteric mTORC1/2 inhibitors may provide a greater degree of inhibition^[36,122]^. As a result, increasing numbers of non-allosteric mTORC1/2 inhibitors, e.g., AZD2014, AZD8055, OSI-027, and INK128/MLN128, have been investigated *in vitro *and *in vivo*. David-West *et al*.^[122]^ demonstrated that platinum-resistant ovarian cancer cells (OVCAR-3) can be re-sensitized to carboplatin by inhibition of mTORC1/2 using INK128/MLN128. Similarly, results from the study using the non-allosteric mTORC1/2 inhibitor vistusertib (AZD2014) showed that the combination treatment of vistusertib and paclitaxel resulted in a significant reduction in tumor growth and increase in apoptosis in a cisplatin-resistant xenograft (A2780cis) model, which led to the initiation of a clinical trial to evaluate this drug combination (NCT02193633)^[123]^. A further study examined the combination of AZD8055 with the PI3K inhibitor GDC0941 and MEK1/2 inhibitor selumetinib in ovarian clear cell carcinoma cell lines and PDX models. Low-dose triple combination of inhibitors reduced kinase activity in both PI3K/AKT/mTOR and mitogen-activated protein kinase pathways, inhibited proliferation *in vitro*, and significantly reduced tumor growth in PDX models, suggesting this combination merits further clinical exploration^[124]^.

Whether non-allosteric mTOR1/2 inhibitors will offer any clinical superiority over allosteric inhibitors remains unclear, and although overall success in ovarian cancer is currently limited, both drug classes continue to be tested in Phase I and II studies. Despite positive trial outcomes with temsirolimus, another Phase II trial investigating the temsirolimus in women with platinum-refractory/resistant ovarian cancer or advanced/recurrent endometrial carcinoma was terminated due to efficacy not meeting the pre-defined levels required as approximately 48% of OC and 40% of EC patients had progressive disease after eight weeks of treatment (NCT00926107)^[125]^. A Phase II trial evaluating the tolerability and activity of the single agent allosteric mTORC1 inhibitor ridaforolimus in women with recurrent endometrial cancer revealed a weak treatment response and significant toxicity (NCT00770185)^[126]^. However, a Phase I open-label trial of the allosteric mTORC1 inhibitor everolimus in combination with the PARP inhibitor niraparib in patients with advanced ovarian and breast cancer is currently recruiting (NCT03154281), as well as a Phase IV study in South Korea (NCT02688881) dispensing sirolimus as a monotherapy in patients with refractory solid tumors (*PIK3CA* mutation, *PIK3CA *amplification, PIK3CA-AKT pathway aberration). Results were recently reported from a first-in-human Phase I study (NCT01353625) in patients with advanced solid (including ovarian) or hematological malignancies for an inhibitor of mTORC1/2 which is also a potent inhibitor of DNA-PK (CC-115), concluding that CC-115 was well-tolerated with toxicities consistent with other mTOR inhibitor treatments, and suggesting that this novel dual mTOR/DNA-PK inhibitor could be a favorable treatment for cancer patients^[106]^.

#### Dual PI3K/mTOR inhibitors

By inhibiting all four PI3K isoforms and both mTORC1 and mTORC2 complexes, the feedback activation of PI3K by mTORC can be overcome resulting in suppression of multiple significant nodes in the PI3K/AKT/mTOR pathway, theoretically leading to a more complete inhibition of the pathway^[36,52,90]^. As dual PI3K/mTOR inhibitors have a comparable poor toxicity profile to pan-PI3K inhibitors, with common adverse events including fatigue, nausea, vomiting and diarrhea, no dual PI3K/mTOR inhibitors have yet advanced for clinical use for any cancer type. Limited numbers of dual PI3K/mTOR inhibitors have progressed to clinical trials in ovarian cancer, despite numerous pre-clinical studies showing positive anti-tumor activity and outcomes. For example, Yuan *et al*.^[127]^ tested the dual pan-PI3K/mTOR inhibitor PF-04691502 as a single agent in ovarian cancer SKOV3 xenograft models and observed dose-dependent reduction in tumor volume in PIK3CA mutant SKOV3 xenografts, which also correlated with plasma concentration, induced time- and dose-dependent target modulation and following oral administration, and was well tolerated without body weight loss. The PI3K/mTOR inhibitor CMG002 was demonstrated to re-sensitize chemo-resistant ovarian cancer cells (paclitaxel-resistant SKpac17 and A2780cis) to chemotherapies *in vitro*, and showed a marked decrease in tumor growth in xenograft mouse models of each cell line, either alone or in combination with paclitaxel or cisplatin^[128]^. Lezzi *et al*.^[129]^ showed that the combined treatment of the dual PI3K/mTOR inhibitor gedatolisib and c-MET inhibitor crizotinib indicated that crizotinib was able to potentiate the activity of gedatolisib in ovarian cell lines (A2780 and SKOV3) and due to favorable tolerability, it could be a potential combination in settings in which either inhibitor alone already had some significant activity. A recent study profiling the anti-tumor effects of 16 different PI3K/AKT/mTOR pathway inhibitors in combination with paclitaxel in a panel of ovarian cancer cell lines and primary ovarian tumor cells covering the four main subtypes of EOC, found that the dual PI3K/mTOR inhibitor GSK458 in particular was a potent inhibitor of proliferation and cell migration* in vitro*, and was able to reduce tumor growth and metastasis in both SKOV3 xenograft and PDCX models *in vivo*^[130]^. Thus, GSK458 was proposed as an attractive candidate for further investigation as a treatment for chemotherapy-resistant ovarian cancer. Deng *et al*.^[131]^ investigated the association of the PI3K/AKT/mTOR pathway with EMT and CSC in EOC platinum resistance, using the dual PI3K/mTOR inhibitor BEZ235. Treatment with BEZ235 and cisplatin inhibited the PI3K/AKT/mTOR pathway, reversed EMT and decreased the expression of CSC markers in platinum resistant A2780cis and IGROV1cis cell lines *in vitro* compared to inhibitor or cisplatin alone, suggesting BEZ235 as a good candidate for treating chemoresistant CSCs^[131]^.

To date only early phase clinical trials have been established for dual PI3K/mTOR inhibitors in ovarian cancer cohorts [Table 2]. The PI3K/mTOR peptide pro-drug SF1126 was investigated in a Phase I first-in-human trial for patients with advanced solid tumors (*n *= 39) including ovarian (*n *= 5). Stable disease in 58% of patients was the best response reached and the pro-drug was found to be well tolerated^[100]^. XL765 (SAR245409) was tested in a first-in-human clinical trial to evaluate safety, maximum tolerated dose, PK, PD and efficacy; stable disease was achieved in 48% of patients with evaluable disease, supporting further development of XL765^[132]^. Other Phase I clinical trials have since been completed with XL765 in solid tumors, including ovarian cancer, and lymphoma, as a monotherapy (NCT01587040; NCT01596270) or in combination with other agents, e.g., erlotinib (NCT00777699), and letrozole (NCT01082068). A Phase II randomized double-blind placebo-controlled trial (NCT01936363) was completed testing the combination MEK1/2 inhibitor (pimasertib) with XL765 in patients with previously treated unresectable borderline or low grade ovarian cancer. Results reported show that while the combination was safe with manageable toxicities for patients, the ORR was 9.4% (80%CI: 3.5-19.7) for the combination treatment, with single agent pimasertib showing an ORR of 12%. The study was terminated due to low ORR and over 50% rate of patients discontinuing the trial^[103]^.

There are a number of on-going Phase I/II trials currently investigating dual-PI3K/mTOR inhibitors in combination with chemotherapy or targeted therapies in breast cancer, renal cell and prostate cancers with promising results that may prove useful for ovarian cancer trials in the future. In particular, the dual PI3K/mTOR inhibitor, LY3023414 (samotolisib), also reported to inhibit DNA-PK, is under investigation in number of Phase I and II trials as a monotherapy in patients with endometrial cancer (NCT02549989), and in combination with chemotherapy or other agents in patients with other solid malignancies, e.g., TNBC trial with prexasertib (NCT04032080), metastatic breast cancer with combination therapies (NCT02057133), and prostate cancer with enzalutamide (NCT02407054)^[52,133,134]^. Due to pre-clinical data showing synergistic inhibition of proliferation in HGSOC cells *in vitro* and significantly enhancing efficacy (*P *< 0.001) in combination compared to single agent in HGSOC OV-90 and Cov504 xenografts^[135]^, a Phase Ib trial (NCT02124148) was established (and recently completed, results pending) to assess the safety of prexasertib with LY3023414 in patients with solid tumors including ovarian. Although there have been multiple promising pre-clinical studies and early phase clinical trials, whether dual PI3K/mTOR inhibitors will advance through later phase clinical trials and into clinical use will depend on overcoming significant toxicities observed with dual PI3K/mTOR pathway inhibitor treatments. Despite being able to overcome unwanted pathway feedback activation, as inhibitors of multiple kinases, the considerable off-target and toxic effects observed may deter further clinical development.

#### AKT inhibitors

Targeting AKT is an attractive therapeutic prospect as inhibiting this key effector node of the PI3K/AKT/mTOR pathway leads to blocking mTORC1 activation and controlling the downstream effects of the pathway signaling cascade^[36,90]^. Several small molecule inhibitors of AKT have been developed to target the three mammalian isoforms of AKT: AKT1, AKT2, and AKT3. Depending on their mechanism of action, AKT inhibitors can be further classified as lipid-based phosphatidylinositol (PI) analogues, ATP-competitive, or allosteric inhibitors^[90,136]^. While most ATP-competitive AKT inhibitors target all isoforms of AKT (pan-AKT inhibitors), a number of allosteric inhibitors were developed in an attempt to selectively target AKT isoforms and have displayed some level of selectivity^[136]^. As increasing numbers of AKT inhibitors are advancing to clinical trial for treatment of advanced solid malignancies, including ovarian cancer, a number of trials in particular have focused on targeting recurrent chemotherapy-resistant ovarian cancer patients based on pre-clinical evidence of efficacy.

The allosteric AKT inhibitor MK-2206 has been investigated in many pre-clinical cancer studies, including ovarian. In particular, MK-2206 was tested in combination with a number of chemotherapy agents and targeted agents (iapatinib and erlotinib), and synergistic cell growth inhibition in combination with cytotoxic drugs in A2780 ovarian cancer cell lines and a reduction in tumor volume in SKOV3 xenografts in combination with iapatinib was observed^[137]^. A further study in multiple ovarian cell lines with differing PI3K or RAS/RAF pathway alterations, RB1 loss and wild-type for the alterations mentioned, demonstrated synergistic effects of MK-2206 and MEK (PD0325901) inhibitors in PI3K- and RAS-activated ovarian cancer cell lines and xenografts^[138]^. MK-2206 has progressed to clinical trials as a monotherapy or in combination with chemotherapy or other targeted agents. A phase II study tested MK-2206 as a monotherapy in a small cohort of patients with recurrent platinum-resistant ovarian, fallopian tube, or peritoneal cancer (NCT01283035). Poor accrual of patients led to early termination of the trial, but preliminary findings suggested that MK2206 was not clinically effective in the patient cohort (selected due to PI3K/AKT pathway alterations)^[110]^. The non-ATP competitive AKT inhibitor TAS-117 has been tested *in vitro* in combination with chemotherapeutic and targeted agents in A2780 ovarian cancer cells and xenografts, with results indicating that TAS-117 effectively enhances the cytotoxic effects of fluorouracil and cisplatin *in vitro*, and improves the anti-tumor effect of carboplatin *in vivo*^[139]^. One of the most studied AKT inhibitors, the PI-analogue perifosine, was tested in combination with paclitaxel in a study by Sun *et al*.^[140]^ demonstrating an increase in ovarian cancer cell apoptosis in the CaOV-3 cell line. Perifosine has been investigated in over 40 clinical trials in multiple cancer types including ovarian (ClinicalTrials.gov). In a Phase I trial (NCT00431054), Fu *et al*.^[112]^ examined the combined effects of perifosine and docetaxel in taxane and platinum-resistant or refractory epithelial ovarian cancer, and reported outcomes of median 1.9 months progression-free survival and 4.5 months overall survival, and no dose-limiting toxicity.

Numerous clinical trials for ATP-competitive pan-AKT inhibitors have been completed showing promising results with more in progress for ovarian cancer and other cancer types [Tables 2 and 3]. The pan-AKT inhibitor uprosertib (GSK2141795) has been studied in a number of dose-escalation trials in combination with other targeted therapies for example with BRAF or MEK inhibitors. A dose-escalation and expansion Phase I study in TNBC and BRSAF-wild type advanced melanoma was terminated early due to a low ORR (< 5%), minimal clinical activity, and the combination was not tolerated^[141]^. A Phase I dose-escalation trial (NCT01266954) in recurrent platinum-resistant ovarian cancer patients (*n *= 12) was established to investigate the PK and PD of repeat escalating doses of uprosertib by 18F FDG-PET. No relationship was observed for dose-response between uprosertib PK and fluoro-deoxyglucose F18 PET PD measures, but single-agent activity was observed with a clinical benefit rate of 27% and 30% CA125 response in the cohort^[108,109]^. A similar ATP-competitive AKT inhibitor afurosertib (GSK2110183) was investigated in a Phase Ib/II dose escalation study in combination with carboplatin and paclitaxel in a recurrent platinum-resistant ovarian cancer cohort, with positive findings of an ORR of 32% by Response evaluation criteria in solid tumors (RECIST) 1.1 and 52% by GCIC CA125 criteria, and a median progression-free survival of 7.1 months^[74]^. The PROFECTA-II (phase II) trial has recently opened and is recruiting patients with platinum-resistant ovarian cancer for combination treatments of afuresertib and paclitaxel (NCT04374630).

The pan-AKT inhibitor capivasertib (AZD5363) was first tested in patients with multiple solid tumor types including *PIK3CA *mutated breast and gynecologic cancers in a Phase I, open-label, first-in-human evaluation study (NCT01226316), and reported that capivasertib was well tolerated and had robust target modulation in tumors^[142]^. More recently capivasertib has been tested in a Phase I trial in combination with the PARP inhibitor olaparib for multiple solid tumors (NCT02338622) based on preclinical studies demonstrating synergy between PARP and PI3K/AKT pathway inhibitors in BRCA1/2 deficient and proficient tumors. In the 64 patients enrolled (*n *= 25 ovarian) capivasertib was well tolerated, overall 44.6% of patients achieved clinical benefit (RECIST1.1 complete response/partial response or stable disease > 4 months), 44% of ovarian patients had a median duration of response of 26.6 weeks (11.3-115), and 8/11 platinum resistant ovarian cancer patients showed clinical benefit^[72]^. In general, several AKT inhibitors are well tolerated and continue to be investigated both *in vitro* and in clinical trials in multiple advanced solid tumors. Data from early phase trials examining combinations of AKT inhibitors with chemotherapy agents and targeted therapies for platinum resistant ovarian cancer patients show positive response rates and clinical outcomes. In particular, findings suggest that the combination of AKT inhibition with platinum-based chemotherapy is effective and durable for patients with platinum resistance, and merits further investigation.

## CONCLUSION AND FUTURE PERSPECTIVES

The last two decades have seen an exponential growth in the number of pre-clinical studies and early phase trials in ovarian cancers, targeting different nodes in the PI3K signaling cascade, greatly increasing our understanding of the dysregulation of PI3K/AKT/mTOR pathway. However, disappointingly to date no inhibitors directed towards the PI3K/AKT/mTOR pathway have progressed to late phase clinical trials for ovarian cancer patients. Significant advances are paramount to accelerate new pathway inhibitors to the clinic including characterizing new potential predictive biomarkers in the pathway, exploring new drug combinations, e.g., DDR inhibitors, cell cycle checkpoint inhibitors, and implementing innovative trial designs. Translational research studies including genomic-, proteomic-, and metabolomic-based analyses, should be incorporated into clinical trial designs to help uncover new clinical biomarkers and further investigations into potential mechanisms of resistance to PI3K pathway inhibitors and the effects of pathway inhibitors on ovarian CSC populations. A recent large phase II trial, the MATCH (Molecular Analysis for Therapy Choice) Screening trial (NCT02465060), was established for patients with advanced refractory solid tumors (including ovarian), lymphomas or multiple myeloma and is an ongoing collaborative effort between the NCI Precision Medicine Initiative and several pharmaceutical companies, aiming to match targeted therapy directed by genetic testing of the patient tumor. As mentioned previously, actionable mutations are rare and variable in ovarian cancer, e.g., *PIK3CA* mutations are present in 20%-46% of clear cell ovarian cancers^[53,55,143]^, but this histological subtype is responsible for approximately 5%-10% of EOC. Therefore the study coordinators suggest that to evaluate whether personalized targeting of tumors is effective for cancers such as ovarian with multiple subtypes, as many patients as possible should be screened to determine whether molecular therapy is a beneficial strategy^[144]^. This multi-study trial aims to enroll up to 6452 patients. Inhibitors of the PI3K/AKT/mTOR pathway such as capivasertib or ipatasertib are indicated for patients with *AKT* mutations, or the PI3Kβ inhibitor GSK2636771 is indicated for patients with *PTEN* mutation, deletion and *PTEN* expression. On a smaller scale, the MyTACTIC trial (NCT04632992) aims to evaluate targeted therapies in patients with advanced solid tumors with genomic alterations or protein expression patterns predictive of response in an open-label Phase II study, and includes GDC-0077 indicated for patients with a positive biomarker result for *PIK3CA* or ipatasertib for patients with a positive biomarker result for either *AKT1/2/3* activating mutation or loss/loss of function of *PTEN*.

As medicine moves more towards personalized care, the results of these types of basket trials will be important for future trial designs, identification and validation of actionable predictive biomarkers associated with clinical activity, and selection of the correct targeted therapies for patients with low-frequency molecular alterations such as the different PI3K pathway aberrations observed in ovarian and other gynecological cancers. Development of new compounds with fewer off-target effects is also necessary to limit toxicities and adverse events for patients, as well as explore new rational combination strategies and dosing schedules. Results from monotherapy trials for inhibitors of different nodes of the PI3K/AKT/mTOR pathway report limited responses to single agent inhibitors, but data from early phase combination trials indicate combination therapies to be more effective than single agent treatments. Targeting other nodes of the PI3K/AKT/mTOR pathway is another emerging field with inhibitors in pre-clinical development against eIF4E, S6K, MNK and PIM^[37]^. A greater understanding of the effects of PI3K pathway inhibitors on the different aspects of the tumor microenvironment is also required, which could open up new avenues for combination treatments with immunotherapies. Positive findings have been observed for PI3Kα or AKT inhibitors in combination with olaparib treatment for recurrent and platinum-resistant ovarian cancer patients^[72,93]^, and further combinations with DDR therapeutics warrant investigation in these patient cohorts. It is becoming increasingly evident that PI3K/AKT/mTOR pathway inhibitors, particularly in combination with other chemotherapeutic and/or targeted therapies, may hold significant promise for the future treatment and outcomes of women with chemotherapy-resistant ovarian cancer.

## DECLARATIONS

### Copyright

© The Author(s) 2021.
